# No Association Between Statin Use and the Prognosis of Endometrial Cancer in Women With Type 2 Diabetes

**DOI:** 10.3389/fphar.2021.621180

**Published:** 2021-05-13

**Authors:** Elina Urpilainen, Anne Ahtikoski, Reetta Arima, Ulla Puistola, Peeter Karihtala

**Affiliations:** ^1^ PEDEGO Research Unit, Medical Research Center Oulu, Department of Obstetrics and Gynecology, Oulu University Hospital and University of Oulu, Oulu, Finland; ^2^ Cancer and Translational Medicine Research Unit, Department of Pathology, Oulu University Hospital and University of Oulu, Oulu, Finland; ^3^ Department of Obstetrics and Gynecology, Central Finland Central Hospital, Jyväskylä, Finland; ^4^ Department of Oncology, University of Helsinki and Helsinki University Comprehensive Cancer Center, Helsinki, Finland

**Keywords:** long-term medication use, cancer, prognosis, statin, type 2 diabetes, endometrial cancer, prognostic factors

## Abstract

Preclinical studies have suggested statins have antiproliferative and anti-metastatic effects on endometrial cancer cells. Similarly, most previous epidemiological studies have reported a better prognosis of endometrial cancer in patients who used statins. In this study, we explored the role of statins in the prognosis of endometrial cancer in women with type 2 diabetes in a hospital-based cohort. This retrospective cohort consisted of 119 women with type 2 diabetes who were diagnosed and treated for endometrial cancer at Oulu University Hospital, Finland, between 2007 and 2014. The patients were classified as statin users (*n* = 58) and nonusers (*n* = 61) based on the type of medication they were using at the time of endometrial cancer diagnosis. Statin use showed no association with progression-free survival or overall survival in the whole cohort nor the subgroups with type I or type II histology, in lower or higher body mass index groups, or at an early or advanced stage. The results remained similar in the multivariate analysis after adjusting for the patient’s age, cancer stage, and histology. Furthermore, statin use seemed not to have any association with most of the prognostic factors at the time of endometrial cancer diagnosis.

## Introduction

Endometrial cancer is the fifth most common cancer among women worldwide (Ferlay et al., 2019) and the incidence is increasing mostly due to obesity (Raglan et al., 2019). Type 2 diabetes (T2D) is a rapidly increasing chronic disease, and it has been estimated that more than 460 million adults have diabetes, with more than 90% suffering from T2D (IDF Diabetes Atlas, 2019). T2D itself seems to be an independent risk factor for endometrial cancer, although these two diseases also share some other common risk factors in addition to obesity (Liao et al., 2014).

Most endometrial cancers are diagnosed at early stages (Morice et al., 2016), and the 5-year survival rate is 95% in early endometrial cancer (Siegel et al., 2019). However, survival decreases to as low as 16% in advanced cancer (Siegel et al., 2019). Traditionally, endometrial cancer is grouped into type I and type II cancers (Bokhman, 1983). Type I endometrial cancers are more common and have a better prognosis (Samarnthai et al., 2010). Type II endometrial cancers are poorly differentiated, they have more commonly a higher frequency of deep myometrial invasion and pelvic lymph node metastases and decreased sensitivity to progesterone (Suarez et al., 2017).

As drug development costs have significantly increased, drug repositioning has become a more attractive option (Kobayashi et al., 2019). Drug repositioning aims to discover new efficacies and practices for existing drugs that have already been demonstrated to be safe in humans and have established pharmacokinetics through clinical use (Kobayashi et al., 2019).

Patients with T2D have an increased risk of both cardiovascular diseases and hypercholesterolemia, and are widely treated with statin therapy. In Finland, 40% of patients diagnosed with T2D have been found to use lipid-lowering medication without diagnosis of coronary heart disease, and the percentage of medication users increases to 73% in patients with coincident coronary heart disease (Vehko et al., 2013). In Finnish national guidelines, the level of low-density lipoprotein in plasma is recommended to be less than 2.5 mmol/L in patients with T2D regardless of other risk factors and less than 1.8 mmol/L in those patients with coincident coronary heart disease, cerebral arterial disease or peripheral arterial disease and if the targets are not met otherwise, statin therapy is recommended to be initiated (Type 2 diabetes: Current Care Guidelines, 2020). Statins are HMG-CoA reductase inhibitors that block the formation of cholesterol by inhibiting the mevalonate pathway. In preclinical studies, statins seem to have antiproliferative and anti-metastatic effect on endometrial cancer cells (Kato et al., 2010; Schointuch et al., 2014). The possible anticancer effects of statins are believed to be derived from the mevalonate pathway, as the pathway produces biologically active metabolites that contribute to tumor-cell proliferation, survival, invasion, and metastasis (Thurnher et al., 2012). Furthermore, the mutant p53 protein, which is present in the majority of all cancers, upregulates the mevalonate pathway supporting the anticancer role of statins (Thurnher et al., 2012). Mutant p53 is common in type II endometrial cancers but rare in type I endometrial cancers (Samarnthai et al., 2010; Banno et al., 2014). Lipophilic statins seem to have more anticancerous effects than hydrophilic statins (Kato et al., 2010). This difference is mainly due to the differences in cell membrane penetration, as lipophilic statins easily diffuse across the membranes, while hydrophilic statins rely on active transport (Beckwitt et al., 2018). In a recent meta-analysis, statin use was associated with both better overall survival (OS) and lower cancer-related mortality (Li et al., 2018). Furthermore, a Finnish register-based study reported lower mortality in non-endometrioid endometrial cancer among statin users in patients with T2D (Arima et al., 2018).

This retrospective study aimed to determine if statin use would improve the prognosis of endometrial cancer in women with T2D in a hospital-based cohort when more data of the possible confounding factors of the patients are available than in traditional register-based studies.

## Materials and Methods

### Study Population

Our study population consisted of all women with T2D who were histological diagnosed with and treated for endometrial cancer at Oulu University Hospital in Finland between 2007 and 2014. Women, whose statin use was not known, were excluded. Data were obtained from the Oulu University Hospital records. Information extracted from the records included the patient’s age at diagnosis of endometrial cancer, parity, statin use, antidiabetic medication, menopause age, the presence of fatty liver, and body mass index (BMI). Cancer-related information such as stage, histology, peritoneal cytology, myometrial invasion, lymphovascular invasion (LVI), estrogen receptor (ER) status, residual tumor after surgery, and adjuvant treatment received was also obtained from the hospital records. All endometrial cancer diagnoses were based on histology, and stages were rechecked and fitted to the current International Federation of Gynecology and Obstetrics (FIGO) stages (Pecorelli, 2009). According to their histologies, the cancers were categorized as type I and type II cancers such that grades 1 and 2 endometrioid endometrial and mucinous cancers were labeled as type I cancers while grade 3 endometrioid, serous, clear cell, mixed, and undifferentiated cancers and carcinosarcomas were classified as type II cancers. Patients were categorized as statin users if they had used statin at the time of endometrial cancer diagnosis or cancer surgery.

The follow-up of the patients began at the time of endometrial cancer surgery, except for patients who were not eligible for surgery (*n* = 14). In those cases, the start of the follow-up was the date of the diagnosis of endometrial cancer in the endometrial biopsy. Follow-up ended at the time of death or closure of the follow-up period (August 7, 2018). The median follow-up time was 63 months.

### Statistical Methods

Statistical analysis was performed using IBM SPSS Statistics, version 25 (IBM Corporation, Armonk, NY, United States) and GraphPad Prism, version 8.0.2 (GraphPad Software, San Diego, CA, United States) software. Comparisons between statin users and nonusers were evaluated using the two-sample *t*-test and Mann–Whitney *U* test for continuous variables and Pearson chi-square and Fisher’s exact tests for categorical variables. The FIGO stages were distributed into two categories—early and advanced. The early stage category included FIGO stages IA and IB, while the advanced stage included stages II, III, and IV. Kaplan–Meier curves with the log-rank test were applied to the survival analysis. Progression-free survival (PFS) was calculated from the time of the surgery to the date of radiological progression. OS was calculated from the time of surgery or cancer diagnosis to the time of death. Cox regression analysis was applied for multivariate analysis—where age at diagnosis, histology, and stage of endometrial cancer, along with statin use, were included in the model. In all the statistical analyses, *p*–values < 0.05 were considered statistically significant.

## Results

In the chosen institution, there were 121 women with T2D diagnosed with endometrial cancer between 2007 and 2014. Two women were excluded from further analysis because of the lack of information on their statin use. The statin user group had 58 women while the statin nonuser group had 61 women. Patient characteristics were similar in both medication groups. The mean age at diagnosis was 71.2 years among the statin users and 70.9 years among statin nonusers (Table 1). Median BMI was 34.0 in statin users and 35.5 in statin nonusers. Menopausal status, parity, antidiabetic medication used, and adjuvant treatment received were similar in both medication groups.

**TABLE 1:** Patient characteristics in statin users and nonusers.

	Statin users (*n* = 58)	Statin nonusers (*n* = 61)	*p*-value
Age at diagnosis (years)			0.86^a^
Mean	71.2	70.9	
STD	1.09	1.21	
Range	53–88	51–88	
Age group at diagnosis			0.19^b^
<65 years	12	19	
≥65 years	46	42	
BMI (kg/m^2^)			0.30^c^
Median	34.0	35.5	
Range	19–51	23–65	
Missing	4	5	
BMI class (kg/m^2^)			0.57^b^
<35	28	26	
≥35	26	30	
Missing			
Parity			0.41^c^
Median	2.7	2.7	
Range	0–8	0–13	
Missing	0	3	
Menopause age			
Premenopausal	0	5	0.07^b^
Under age 50	5	10	
50–53	27	22	
≥54	15	14	
Missing	11	10	
Fatty liver			0.66^b^
Yes	22	19	
No	20	21	
Missing	16	21	
ADM			
Metformin ± other oral ADM	32	26	0.33^b^
Insulin ± oral ADM	17	18	
Other oral ADM alone	2	6	
None	7	11	
Adjuvant treatment			0.45^d^
None	26	30	
WPRT	10	12	
Chemotherapy	11	8	
Vaginal brachytherapy	7	5	
Intracavitary radiation	2	6	
Hormonal treatment	2	0	

a Student’s *t*-test.

b Pearson’s chi-square test.

c Mann-Whitney *U* test.

d Fisher’s exact test.

STD, standard deviation; BMI, body mass index; ADM, Antidiabetic medication; WPRT, Whole-Pelvic Radiation Therapy.

Tumor characteristics were also quite similar in both medication groups (Table 2). Most indicators of poor prognoses, such as advanced stage, type II histology, and deep myometrial invasion, were similar in both medication groups. However, the presence of LVI was more common in statin users than nonusers (*p* = 0.028). Since most of the endometrial cancers were endometrioid, ER was mostly positive in both medication groups and showed no statistically significant difference. In addition, peritoneal cytology and residual tumor after surgery were similar in statin users and nonusers.

**TABLE 2:** Tumor characteristics in statin users and nonusers.

	Statin users (*n* = 58)	Statin nonusers (*n* = 61)	*p*-value
Histology			0.56^a^
Type I	42	47	
Type II	16	14	
Stage			0.33^a^
Early (IA–IB)	38	44	
Advanced (≥ II)	17	13	
Missing	3	4	
Deep MI			
Yes	20	21	0.66^a^
No	34	30	
Missing	4	10	
LVI			
Yes	24	12	0.028^a^
No	29	37	
Missing	5	12	
ER status			
Positive	48	50	0.77^a^
Negative	10	9	
Missing	0	2	
Peritoneal cytology			
I–II	41	41	0.14^b^
III	0	4	
IV	4	1	
V	2	2	
Missing	11	13	
Residual tumor			
No	53	46	0.11^b^
Yes	1	3	
Missing	0	2	
No surgery	4	10	

a Pearson’s chi-square test.

b Fisher’s exact test.

MI, myometrial invasion; LVI, lymphovascular invasion; ER, estrogen receptor.

**FIGURE 1:**
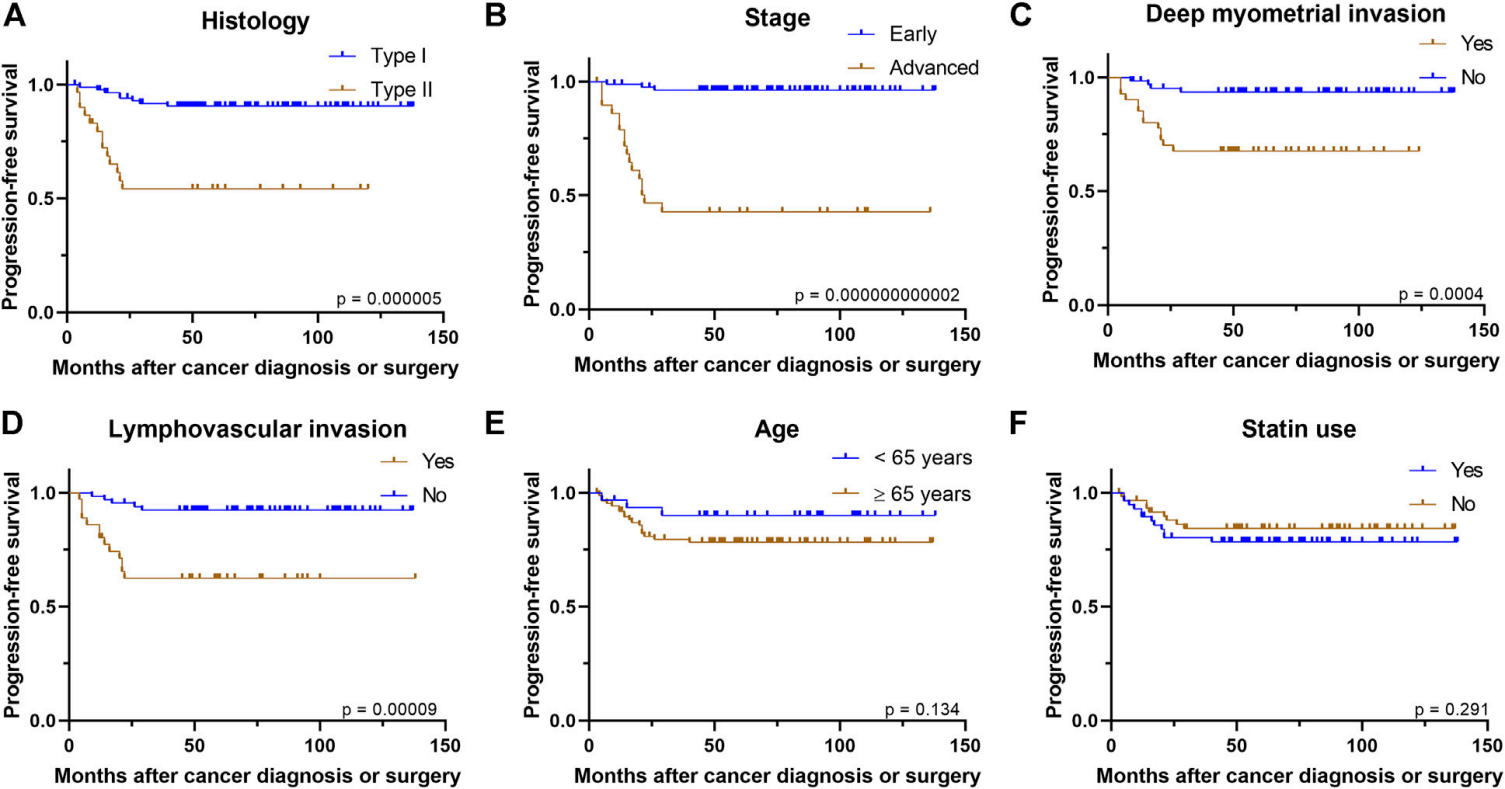
Kaplan–Meier curves demonstrate associations between **(A)** histology **(B)** stage **(C)** deep myometrial invasion **(D)** presence of lymphovascular invasion **(E)** age, or **(F)** statin use and progression-free survival.

As expected, PFS was notably worse in more advanced cancer stage (*p* = 0.000000000002), type II histology (*p* = 0.000005), deep myometrial invasion (*p* = 0.0004) and presence of LVI (*p* = 0.00009) in univariate analysis (Figure 1). Older age (*p* = 0.13), BMI group (*p* = 0.54) or ER status of the tumor (*p* = 0.35) had no association with PFS.

In the whole study cohort, statin use seemed not to have association with PFS (*p* = 0.29) in univariate analysis. In addition, the results remained similar in the subgroup analysis, as statin use was not associated with PFS in type I histology (*p* = 0.88), type II histology (*p* = 0.20), early stage (*p* = 0.67), advanced stage (*p* = 0.70), superficial myometrial invasion (*p* = 0.36), deep myometrial invasion (*p* = 0.26), presence of LVI (*p* = 0.92), absence of LVI (*p* = 0.87) (Supplementary Figure S1), ER-positive cancers (*p* = 0.25), ER-negative cancers (*p* = 0.87), higher BMI group (*p* = 0.70), lower BMI group (*p* = 0.09), younger age group (*p* = 0.85) or older age group (*p* = 0.30). In Cox regression analysis, the advanced stage was the only variable associated with poorer PFS after adjusting for statin use, histology, and patient’s age (Table 3).

**TABLE 3:** The results of multivariate analysis.

	Overall survival			Progression-free survival		
	HR	95% CI	*p*-value^a^	HR	95% CI	*p*-value^a^
Stage	2.173	1.01–4.69	0.048	15.842	4.21–59.53	0.00004
Histology	3.477	1.64–7.38	0.001	2.438	0.86–6.95	0.095
Age	1.063	1.02–1.11	0.009	0.995	0.94–1.06	0.858
Statin use	0.995	0.50–2.00	0.998	0.995	0.40–2.50	0.992

a Cox regression analysis.

HR, hazard ratio; CI, confidence interval.

**FIGURE 2:**
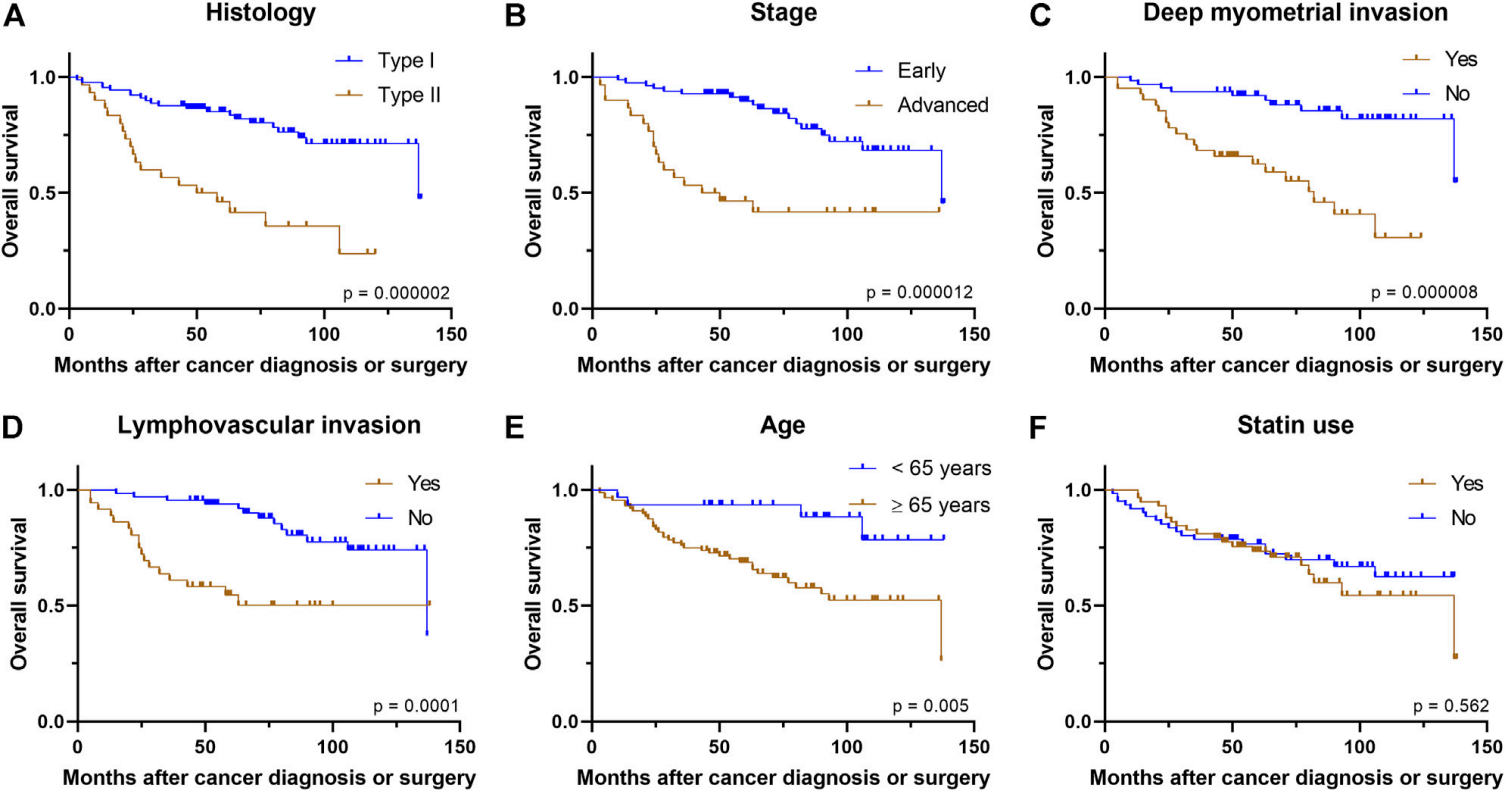
Kaplan–Meier curves demonstrate associations between **(A)** histology **(B)** stage **(C)** deep myometrial invasion **(D)** presence of lymphovascular invasion **(E)** age, or **(F)** statin use and overall survival.

Similar to PFS, univariate analysis showed that OS was worse in those with type II histology (*p* = 0.000002), advanced stage (*p* = 0.000012), older age (*p* = 0.005), deep myometrial invasion (*p* = 0.000008), and the presence of LVI (*p* = 0.0001) (Figure 2). However, patient’s BMI at the time of diagnosis (*p* = 0.94) and ER status of the tumor (*p* = 0.77) showed no association with OS.

Statin use was not associated with OS in the univariate analysis of the whole study population (*p* = 0.56). Similarly, statin use did not have association with OS in subgroups of type I histology (*p* = 0.55), type II histology (*p* = 0.67), early stage (*p* = 0.34), advanced stage (*p* = 0.38), superficial myometrial invasion (*p* = 0.22), deep myometrial invasion (*p* = 0.59), presence of LVI (*p* = 0.69), absence of LVI (*p* = 0.73) (Supplementary Figure S2), lower (<35 kg/m^2^) BMI class (*p* = 0.81), higher (≥35 kg/m^2^) BMI class (*p* = 0.91), younger age group (*p* = 0.57), older age group (*p* = 0.97), ER-positive cancers (*p* = 0.44), or ER-negative cancers (*p* = 0.75), Furthermore, Cox regression analysis showed that statin use was not associated with OS after adjusting for histology type, stage and patient’s age [Hazard ratio (HR) 0.995, 95% confidence interval (CI): 0.50–2.00; Table 3].

## Discussion

In our study, statin use did not have an association with PFS or OS in patients with T2D diagnosed with endometrial cancer either in the whole population or in the subgroups. In addition, statin use showed no association with major prognostic factors of endometrial cancer at the time of cancer diagnosis, although the presence of LVI was more common among the statin users. Two thirds of statin users had LVI in their tumors, compared to 44% of LVI in nonuser group. Due to relatively low sample sizes in both groups, confirmatory studies with larger material are warranted.

Most previous studies concerning statin use and prognosis of endometrial cancer have not focused on women with T2D. The only previous study which focused solely on women with T2D was our study in which statin use was associated with lower mortality from non-endometrioid endometrial cancer in a nationwide database consisted of patients with T2D (Arima et al., 2018). The proportion of women with T2D in previous studies explored statin use have varied from 13 to 38% (Nevadunsky et al., 2015; Yoon et al., 2015; Feng et al., 2016; Sperling et al., 2018; Segev et al., 2020). The number of women with T2D has not been reported in all studies (Lavie et al., 2013; Sanni et al., 2017).

When assessing all the cancers, patients with diabetes have lower OS and might be treated less aggressively probably due to comorbidities (van de Poll-Franse et al., 2007). However, the presence of diabetes did not seem to have an association with the treatment received for endometrial cancer (van de Poll-Franse et al., 2007). Patients with diabetes who are diagnosed with endometrial cancer have increased all-cause mortality compared to women without diabetes but this difference was not seen in cancer-related mortality (Chia et al., 2007). Although higher BMI (≥35 kg/m^2^) was not linked with poorer OS or PFS in our study cohort, in a meta-analysis higher BMI (≥40 kg/m^2^) was linked with increased all-cause mortality in patients with endometrial cancer (Secord et al., 2016). In addition, a previous study has reported BMI to be associated with increased mortality from both all-causes and endometrial cancer, while the presence of diabetes was found to increase cancer-related mortality, especially among non-obese women in both endometrioid and non-endometrioid histologies (Nagle et al., 2018). Furthermore, women diagnosed with endometrial cancer have increased cardiovascular mortality compared to the general population (Felix et al., 2017).

Similarly to our study, some previous studies did not observe any association between statin use and better prognosis in endometrial cancer when all histologies were analyzed together (Yoon et al., 2015; Sanni et al., 2017; Segev et al., 2020). However, some other studies have reported lower mortality from endometrial cancer in type II or non-endometrioid histology (Nevadunsky et al., 2015; Feng et al., 2016; Arima et al., 2018), while Sperling et al. (2018) observed decreased mortality from endometrial cancer and other causes in statin users in both endometrioid and non-endometrioid histologies. Furthermore, Lavie et al. (2013) found better OS, irrespective of the histology in patients who used statin only after an endometrial cancer diagnosis. As previous studies suggest, statin’s beneficial effect in endometrial cancer might be seen in only type II histology. However, we were unable to analyze these two histology groups separately due to the small number of type 2 histology (*n* = 30) in our cohort.

There are differences in mechanisms in cancer development in type I and type II endometrial cancers (Banno et al., 2014), which might also have an impact on treatment choices and prognosis. Type I endometrial cancers are more common in premenopausal or perimenopausal women and develop in an estrogen-depend manner from atypical endometrial hyperplasia (Banno et al., 2014). Therefore, those tumors are usually positive for both estrogen and progesterone receptor and well-differentiated with less frequent lymph node metastases, less myometrial invasion and better prognosis (Banno et al., 2014). Contrary, type II endometrial cancer develops more commonly among postmenopausal women and estrogen-independently via *de novo* carcinogenesis from normal endometrium without precancerous lesions (Banno et al., 2014). Gene mutations associated with type I endometrial cancer include mutations in tumor suppressor gene *PTEN*, *E-cadherin* component *β-catenin* and *K-ras oncogene*, while gene mutations in oncogene *HER2* and tumor suppressor gene p53 are seen in type II endometrial cancers (Banno et al., 2014). The common occurrence of p53 mutations in type II endometrial cancer could explain the favorable effect of statins (Kobayashi et al., 2019).

Our cohort can be considered generally representative in terms of prognostic factors, as type II histology, advanced cancer stage, deep myometrial invasion, presence of LVI, and older age were all poor prognostic factors in OS analysis. Similarly, type II histology, advanced cancer stage, deep myometrial invasion, and presence of LVI predicted poorer PFS. Stage, the most prominent prognostic factor of endometrial cancer, remains the only significant prognostic factor in PFS after adjusting for histology, age, and statin use.

Survival of patients with endometrial cancer in our study was relatively low as only 66.4% of the patients were alive at the end of our follow-up. In contrast, the average 5-year survival among patients with endometrial cancer in Finland was reported as 81.4% (Finnish Cancer Registry, 2020). The lower survival in our study population is mainly because patients with T2D have more coincident diseases than the average population. This is consistent with a previous study where the overall mortality in endometrial cancer patients with pre-existing diabetes was found to be high (Barone et al., 2008), although the association between cancer-related mortality and diabetes was not robust (Tsilidis et al., 2015).

The strengths of this study include reliable and precise data on patient characteristics, cancer characteristics, and over five-year median follow-up. We had data on the patient at the time of endometrial diagnoses, such as BMI and parity, and cancer-related data such as ER status and myometrial invasion, which are usually lacking in register-based studies. However, information on the cause of death was not available and the sample size was rather small due to the single-institution based data.

Weaknesses of this study are the lack of information on the duration and severity of diabetes along with the dose of the medications used, which might lead to a bias, as a longer duration of diabetes is associated with increased cardiovascular mortality, among other comorbidities (Zoungas et al., 2014). Information on the cause of death was not available in the hospital records. In addition, the cholesterol levels of the patients and whether the goals in hypercholesterolemia treatment were met is not known in our study. It seems that hypercholesterolemia itself is also associated with neoplasia progression and dedifferentiation and by reducing LDL cholesterol with diet or drugs it is plausible to reduce endothelial activation and prevent interplay between the inflammatory mediators (Mehta et al., 1997). Although we had no information on the statin type that the patients used, it is known that the majority of Finnish patients use lipophilic statins, mostly simvastatin and atorvastatin (Arima et al., 2017). We lacked the data of the duration and dose of statin therapy. Statin users used statins at the time of endometrial cancer surgery or at time of endometrial cancer diagnosis in those cases who were not eligible for surgery. In this study, we presumed continuous statin exposure after the cancer diagnosis, though we could not verify the duration of statin use through prescription data. However, in the literature concerning breast cancer prognosis, it has been observed that prediagnostic statin users are most likely to continue statin use after cancer diagnosis (Borgquist et al., 2019).

The current knowledge from *The Cancer Genome Atlas* defined four clinically distinct endometrial cancer types based on their p53 mutational burden, exonuclease domain of the DNA polymerase epsilon (POLE) mutations and microsatellite instability (Cancer Genome Atlas Research Network et al., 2013; Stelloo et al., 2016). We were unable to recategorize our endometrial cancer cases according to *The Cancer Genome Atlas* as POLE mutation analysis is not yet available in every-day cancer diagnostics and microsatellite instability (MSI) status was not analyzed in the primary diagnostics in our patient group. Lack of information on p53 mutation status in our study is probably a minor limitation since most grade 3 endometrioid carcinomas with p53 mutation would anyway have a poor prognosis and belong to the type II cancer group.

To conclude, statin use was not associated with PFS or OS in patients with T2D diagnosed with endometrial cancer. Furthermore, statin use seemed not to have any association with most of the prognostic factors at the time of endometrial cancer diagnosis.

## Data Availability

The raw data supporting the conclusions of this article will be made available by the authors, without undue reservation.
