# Supplementary figures and images for: No Association Between Statin Use and the Prognosis of Endometrial Cancer in Women With Type 2 Diabetes
Source: Front Pharmacol. 2021 May 13;12:621180. doi: 10.3389/fphar.2021.621180 (PMC8155720; doi:10.3389/fphar.2021.621180)

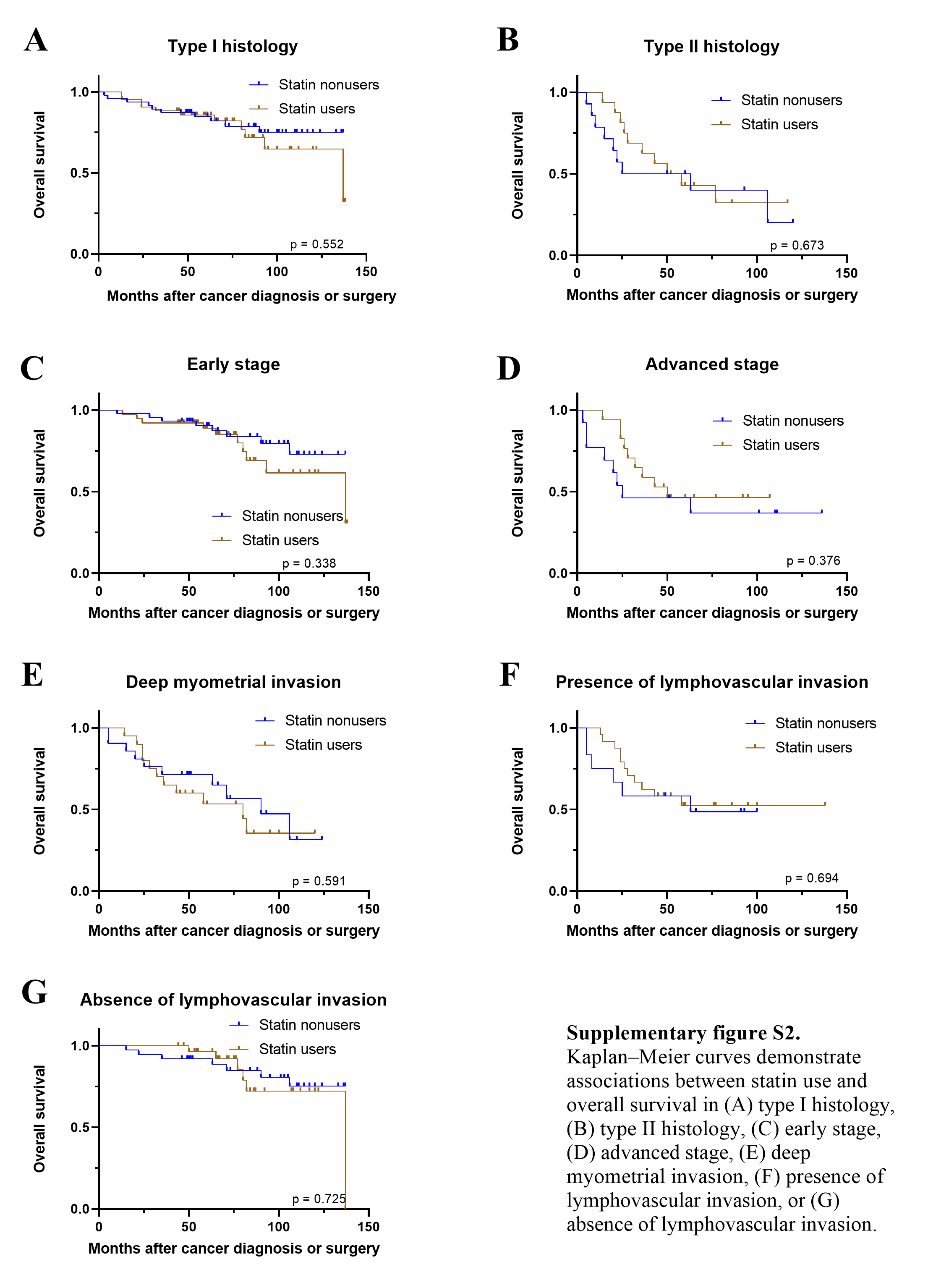

Supplement: Supplementary file 1 [file Image2.tif]

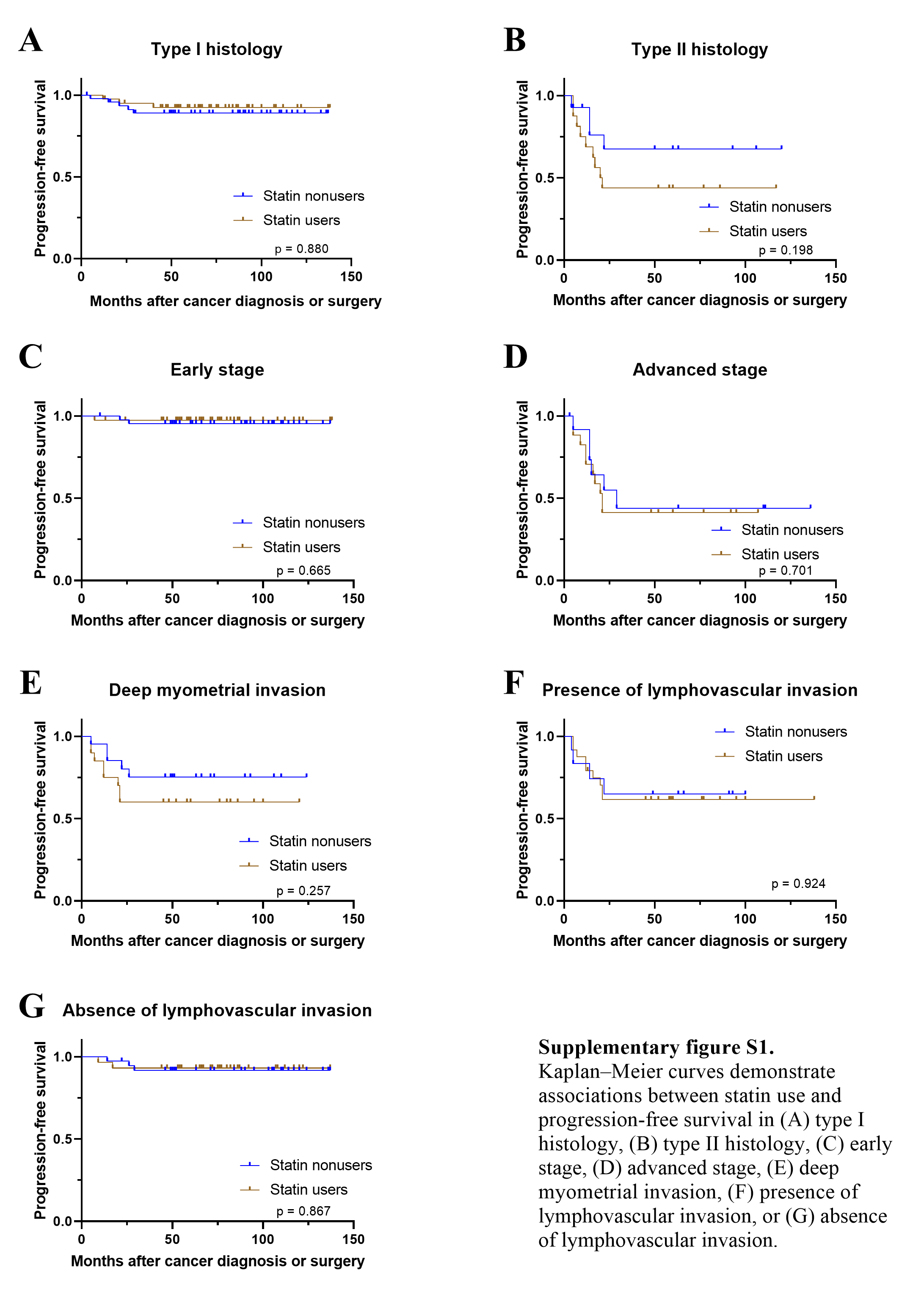

Supplement: Supplementary file 2 [file Image1.tif]
